# A dose-escalating toxicology study of the candidate biologic ELP-VEGF

**DOI:** 10.1038/s41598-021-85693-6

**Published:** 2021-03-18

**Authors:** Jamarius P. Waller, Stephen P. Burke, Jason Engel, Alejandro R. Chade, Gene L. Bidwell

**Affiliations:** 1 Department of Pharmacology and Toxicology, University of Mississippi Medical Center, 2500 North State Street, Jackson, MS 39216 USA; 2 Department of Neurology, University of Mississippi Medical Center, 2500 North State Street, Jackson, MS 39216 USA; 3 Department of Physiology and Biophysics, University of Mississippi Medical Center, 2500 North State Street, Jackson, MS 39216 USA; 4 Department of Medicine, University of Mississippi Medical Center, 2500 North State Street, Jackson, MS 39216 USA; 5 Department of Radiology, University of Mississippi Medical Center, 2500 North State Street, Jackson, MS 39216 USA; 6 Department of Cell and Molecular Biology, University of Mississippi Medical Center, 2500 North State Street, Jackson, MS 39216 USA

**Keywords:** Peptides, Drug delivery, Pharmaceutics, Toxicology

## Abstract

Vascular Endothelial Growth Factor (VEGF), a key mediator of angiogenesis and vascular repair, is reduced in chronic ischemic renal diseases, leading to microvascular rarefaction and deterioration of renal function. We developed a chimeric fusion of human VEGF-A_121_ with the carrier protein Elastin-like Polypeptide (ELP-VEGF) to induce therapeutic angiogenesis via targeted renal VEGF therapy. We previously showed that ELP-VEGF improves renal vascular density, renal fibrosis, and renal function in swine models of chronic renal diseases. However, VEGF is a potent cytokine that induces angiogenesis and increases vascular permeability, which could cause undesired off-target effects or be deleterious in a patient with a solid tumor. Therefore, the current study aims to define the toxicological profile of ELP-VEGF and assess its risk for exacerbating tumor progression and vascularity using rodent models. A dose escalating toxicology assessment of ELP-VEGF was performed by administering a bolus intravenous injection at doses ranging from 0.1 to 200 mg/kg in Sprague Dawley (SD) rats. Blood pressure, body weight, and glomerular filtration rate (GFR) were quantified longitudinally, and terminal blood sampling and renal vascular density measurements were made 14 days after treatment. Additionally, the effects of a single administration of ELP-VEGF (0.1–10 mg/kg) on tumor growth rate, mass, and vascular density were examined in a mouse model of breast cancer. At doses up to 200 mg/kg, ELP-VEGF had no effect on body weight, caused no changes in plasma or urinary markers of renal injury, and did not induce renal fibrosis or other histopathological findings in SD rats. At the highest doses (100–200 mg/kg), ELP-VEGF caused an acute, transient hypotension (30 min), increased GFR, and reduced renal microvascular density 14 days after injection. In a mouse tumor model, ELP-VEGF did not affect tumor growth rate or tumor mass, but analysis of tumor vascular density by micro-computed tomography (μCT) revealed significant, dose dependent increases in tumor vascularity after ELP-VEGF administration. ELP-VEGF did not induce toxicity in the therapeutic dosing range, and doses one hundred times higher than the expected maximum therapeutic dose were needed to observe any adverse signs in rats. In breast tumor—bearing mice, ELP-VEGF therapy induced a dose-dependent increase in tumor vascularity, demanding caution for potential use in a patient suffering from kidney disease but with known or suspected malignancy.

## Introduction

Chronic kidney disease (CKD) affects about 15% of the population in the United States^1^. This disease is characterized by a progressive reduction in renal function (mostly secondary to hypertension and diabetes)^1, 2^ and is associated with increased cardiovascular risk. A universal pathological feature of CKD, regardless of etiology, is the presence of progressive renal microvascular rarefaction^3^, which plays an important role in disease progression and outcomes.

Microvascular rarefaction is a functional and/or anatomical loss of microvessels in a given organ or tissue^3^. The etiology is complex and multifactorial, but data suggest that microvascular rarefaction coincides with a reduction in levels of the pro-angiogenic vascular endothelial growth factor (VEGF)^4^. The VEGF family consists of different isoforms. The most studied of these isoforms is VEGF-A because of its role in promoting angiogenesis and vascular repair. Previous studies have shown that, in swine models of renovascular disease (RVD) and CKD, renal bioavailability of VEGF-A decreased as microvascular rarefaction increased^5–7^, which was largely offset by intra-renal replenishment of VEGF^8^ and gave credence to VEGF as a potential therapeutic in the setting RVD.

Recombinant VEGF-A is a protein that is rapidly cleared from the body. A clinical trial examinging recombinant human VEGF-A_165_ for therapeutic angiogenesis revealed that exogenously administered VEGF has a half-life of ~ 30 min following intravenous infusion in humans^9, 10^. In order to extend the half-life of VEGF-A and improve its potential as a therapeutic agent, our lab developed a chimeric protein by fusing human VEGF-A_121_, a non-heparin binding, freely diffusible isoform^11^, to a biopolymer known as elastin-like polypeptide (ELP)^9^. ELP is a polymer derived from a repeat sequence found in the human elastin protein^12^ and possesses many unique properties which aid in the purification and delivery of attached therapeutics^13^. Briefly, ELP fusion to small proteins or peptides increases plasma half-life^14^, reduces immunogenicity^15^, and, in the case of therapeutics for renal disease, improves deposition in the kidney after systemic administration^16, 17^. The fusion protein between ELP and VEGF-A_121_ (referred to subsequently as “ELP-VEGF”) was previously tested in swine models of RVD^18, 19^ and CKD^7^, showing a 13.5 h plasma half-life and improved renoprotection compared to unconjugated recombinant VEGF. The therapeutic effects (improved renal hemodynamics and microvascular integrity, reduced renal injury) were achieved using a single intra-renal dose of 0.1 mg/kg^7, 18^ and a systemic intravenous dose of 1.0 mg/kg^19^. These prior studies established the potential of ELP-VEGF for renal therapeutic angiogenesis and defined therapeutic dosing in a range of 0.1–1.0 mg/kg.

However, there are many disease processes where off-target angiogenesis and proliferation may be less desirable. One example of this is seen in the setting of tumor growth and metastasis. Tumor neovascularization may sustain and even promote tumor progression^20^, and VEGF has been shown to play a central role^21–23^. In addition to promoting angiogenesis, VEGF also stimulates nitric oxide synthase production through activation of the Flk-1 receptor^24^. Therefore, direct VEGF-A administration, even targeted using drug-delivery technology, could potentially cause off-target vascular effects in a multitude of organ systems. Therefore, we felt it important to extend our previous studies to increase the understanding of what other effects may accompany the therapeutic benefit of ELP-VEGF therapy. To accomplish this, we performed a dose escalating toxicology study in healthy Sprague Dawley (SD) rats as well as a tumor progression study in an athymic nude mouse xenograft model of triple-negative breast cancer. Breast cancer was chosen because the two most common cancers in the older patient population who are most likely to be treated for CKD are breast and prostate cancers in women and men, respectively^25^. Since breast cancer has the highest incidence of all cancers, we chose to start with a breast cancer xenograft model in athymic nude mice.

## Results

### Characterization of the ELP-VEGF temperature-induced phase transition

ELP and ELP-fusion proteins undergo a reversible, temperature dependent phase transition and coacervation^26^. The transition temperature (T_t_) of the protein depends on the size of the ELP moiety^27, 28^, the hydrophobicity of the “x” residue in the VPGxG repeat^29^, and the solution conditions^30^, among other factors. Because the ELP-VEGF fusion protein was designed to be used as a soluble biologic, it was built using an ELP moiety with a T_t_ high enough above body temperature to avoid coacervation and aggregation in vivo, but low enough to allow for induced aggregation under high salt conditions to facilitate its purification by inverse transition cycling. However, ELP-VEGF accumulates to very high concentrations in the kidney^16, 18^, and the urine can be concentrated to very high osmolarity in the medullary portions of the tubules and collecting duct under certain physiological conditions^31^, both conditions under which the T_t_ of ELP-VEGF could be reduced. Therefore, the T_t_ of ELP-VEGF, which was previously determined to be 50.4 °C in physiological buffer at a concentration of 10 μM^17^, was determined under a range of ELP-VEGF and NaCl concentrations. The T_t_ of ELP-VEGF was not concentration dependent within the concentration range of 1–100 μM under physiological salt concentrations in PBS (Supplementary Fig. 1a,b). The T_t_ of ELP-VEGF was dependent on the NaCl concentration within the range of osmolarity possible within the urine as it traffics through the tubular system (50–1400 milliosmolar^31^, which was mimicked in vitro using phosphate buffer with NaCl concentrations ranging from 25 to 700 mM). However, even at the highest osmolarity possible within the urine, the T_t_ of ELP-VEGF remained above body temperature (Supplementary Fig. 1c,d).

**Figure 1:**
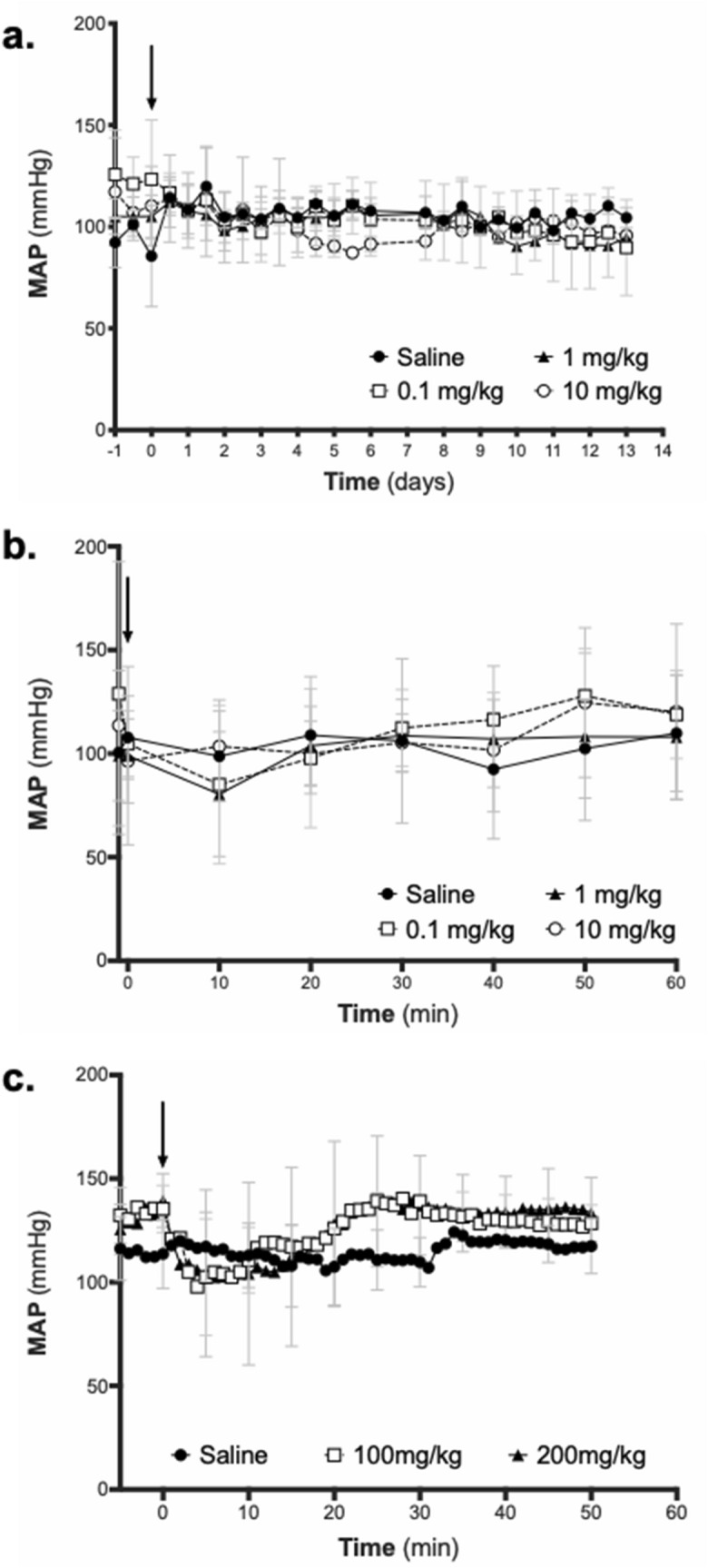
Effect of ELP-VEGF on mean arterial pressure (MAP). Increasing IV bolus doses of ELP-VEGF beginning at the therapeutic doses of 0.1 and 1.0 mg/kg and increasing to the supra-therapeutic dose of 10.0 mg/kg were administered to rats, and MAP was continuously monitored by carotid telemetry for 14 days **(a)**. Acute effects during the first 60 min after injection are shown in **(b)**. Ultra-high doses (100 and 200 mg/kg) were also tested for acute effects on MAP via conscious carotid catheter-based blood pressure monitoring **(c)**. (↓ indicates time of injections).

### Effects of ELP-VEGF on body weight and blood pressure in rats

Mean arterial pressure (MAP) was measured by direct carotid telemetry after intravenous injection of ELP-VEGF at doses of 0.1, 1.0, and 10.0 mg/kg. Analysis of 12-h averages of MAP showed that ELP-VEGF did not affect blood pressure after a single injection at doses up to 10 mg/kg (F (3,12) = 0.5613; p = 0.65), and blood pressure in all groups was unchanged throughout the 14-day study (Fig. 1a). Even when the time immediately after injection was monitored closely by calculating MAP every 10 min using the telemetry data, there was no effect of ELP-VEGF on blood pressure (F (3,14) = 0.16; p = 0.92) (Fig. 1b). A previous study in swine, however, did show a transient decrease in blood pressure after systemic ELP-VEGF administration^19^, which was possibly driven by systemic vasodilation by endothelial VEGF receptor activation^32^. Therefore, to pursue this further, we utilized even higher doses of ELP-VEGF in a separate cohort of rats with acute, conscious carotid artery blood pressure monitoring. In these rats, doses of 100 or 200 mg/kg appeared to cause a transient decrease in blood pressure of approximately 30 mmHg immediately after injection. However, these results did not reach statistical significance (p value for time factor = 0.057). Even after these supratherapeutic doses, the trend for lowered blood pressure disappeared and blood pressure was completely normalized within 30 min after treatment (Fig. 1c).

**Figure 2:**
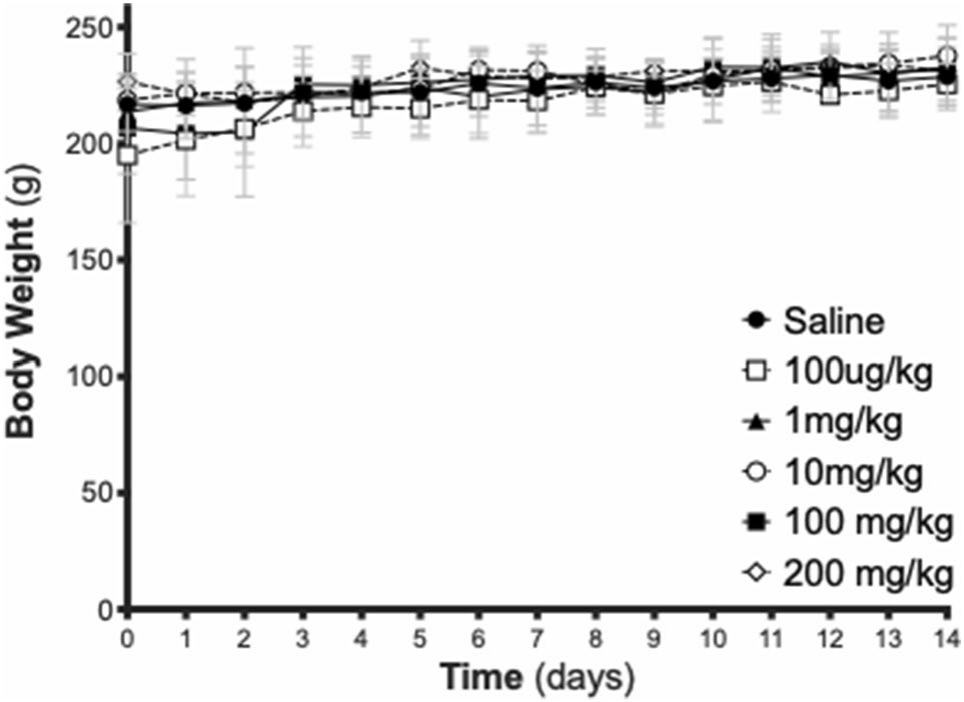
Effect of ELP-VEGF on body weight. Daily body weights of rats are shown after bolus IV injection of ELP-VEGF at increasing doses on Day 0.

For all subsequent analyses, the five dosing groups were pooled since, other than the method of blood pressure monitoring, all other experimental conditions were identical. Daily monitoring of body weight revealed that ELP-VEGF had no effect on body weight at any time during the 14-day protocol at any dose up to 200 mg/kg (F (5,20) = 0.31; p = 0.90) (Fig. 2).

### Effects of ELP-VEGF on renal function

**Figure 3:**
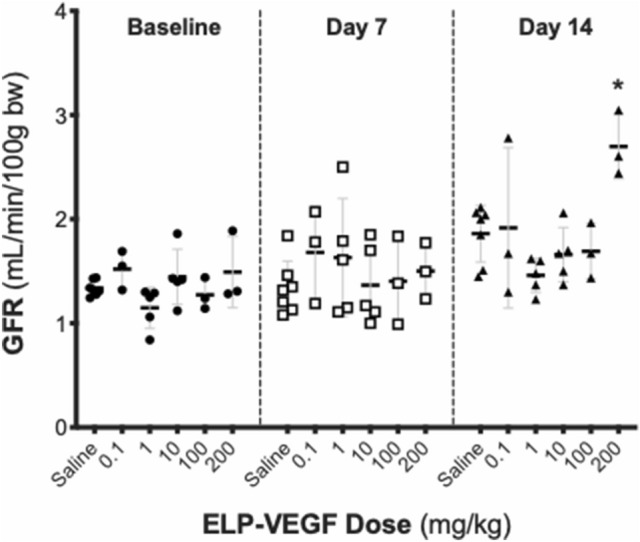
Effect of ELP-VEGF on Renal Function. Glomerular filtration rate (GFR) was assessed by transcutaneous measurement of FITC-sinistrin clearance prior to treatment and 7 and 14 days after treatment with ELP-VEGF at increasing doses. * Statistically significant increase relative to saline treated rats, p < 0.05, two-way ANOVA with post-hoc Dunnett correction for multiple comparisons.

Glomerular Filtration Rate (GFR) was measured by monitoring FITC-sinistrin clearance via a transdermal probe following intravenous injection. There was a significant effect of ELP-VEGF dose on GFR (F (5,60) = 3.31; p = 0.01). Post-hoc analysis revealed that, at doses up to 100 mg/kg, ELP-VEGF had no significant effect on GFR throughout the 14-day post injection period (Fig. 3). However, at the highest dose tested (200 mg/kg), ELP-VEGF injection caused a significant increase in GFR at 14 days post-treatment in comparison to all other doses tested (Dunnett’s corrected p = 0.0021).

**Figure 4:**
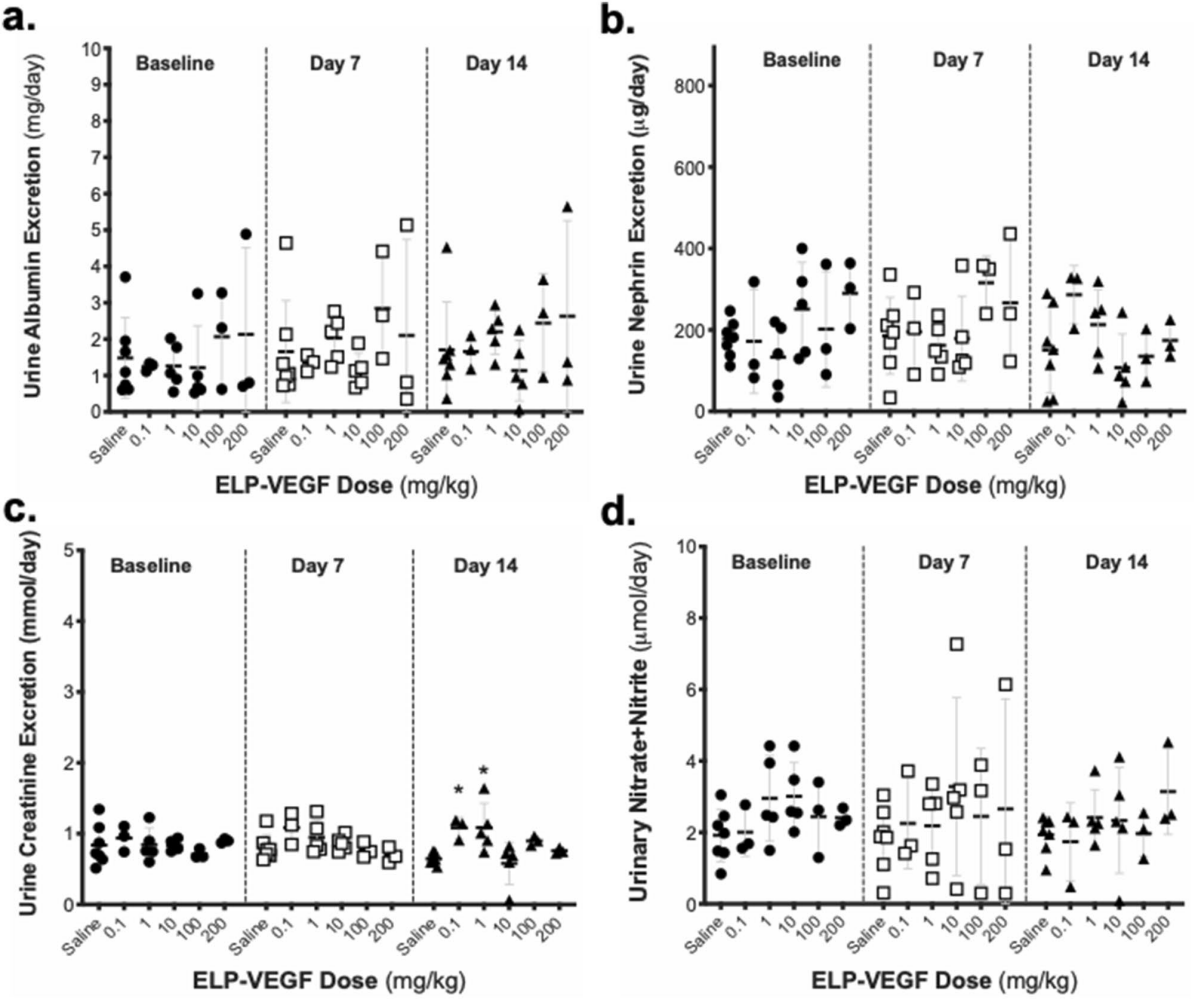
Effect of ELP-VEGF on Urinary Markers of Renal Function and Injury. 24-h urine samples were collected by metabolic cage housing prior to treatment and 7 and 14 days after treatment with ELP-VEGF at increasing doses. **(a)** Albumin excretion and **(b**) urinary nephrin excretion were determined by ELISA to assess renal injury. **(c)** Urine creatinine excretion was determined using a Vet AXCEL chemistry analyzer, and **(d)** urinary nitrate/nitrite excretion was determined by chromogenic assays to assess nitric oxide production. * Statistically significant increase relative to saline treated rats, two-way ANOVA with post-hoc Dunnett correction for multiple comparisons.

In addition to measuring GFR, 24-h urine samples were collected at the same pre- and post-treatment time points for assessment of urinary markers of renal function and injury. No change was observed among any of the treatment doses or time points in urine albumin and nephrin excretion rates (Fig. 4a,b) (albumin F (5,60) = 1.72; p = 0.14) (nephrin F (5,60) = 1.29; p = 0.28). Urine creatinine was mildly elevated on day 14 after ELP-VEGF injection (F (6,60) = 4.19; p = 0.003) at the 0.1 mg/kg dose (Dunnett’s corrected p = 0.01) and 1.0 mg/kg dose (Dunnett’s corrected p = 0.002) (Fig. 4c). However, the urine creatinine values remained within normal limits, and levels were not elevated following higher ELP-VEGF doses (Dunnett’s corrected p = 0.98, 0.27, and 0.91 at 10, 100, and 200 mg/kg, respectively). Urinary nitrate and nitrite, surrogates for renal nitric oxide levels and which we have shown previously are acutely increased by ELP-VEGF treatment^33^, were unchanged on days 7 and 14 after ELP-VEGF administration at all doses tested (F (5,60) = 0.08; p = 0.99) (Fig. 4d).

### Effects of ELP-VEGF on plasma markers of renal and liver injury

Plasma samples collected 14 days after ELP-VEGF injection were analyzed using standard liver and kidney function assays. All liver function measures (Alanine Transaminase (ALT) (F (5,20) = 1.17; p = 0.36), plasma albumin (F (5,20) = 1.05; p = 0.42), and total bilirubin (F (5,20) = 1.04; p = 0.42)) were unchanged by ELP-VEGF treatement (Supplementary Table 1 and Supplementary Fig. 2) with the exception of Aspartate Transaminase (AST) (F (5,20) = 2.90; p = 0.04), which was mildly but statistically significantly elevated (Dunnett’s corrected p = 0.02) at the lowest ELP dose. However, the mild AST elevation following the 0.1 mg/kg ELP-VEGF dose remained within normal limits for AST reference values, and AST was not elevated following the higher ELP-VEGF doses (Dunnett’s adjusted p values = 0.99, 0.73, 0.99, and 0.99 for doses of 1, 10, 100, and 200 mg/kg, respectively, Supplementary Fig. 2). Renal function markers (Blood urea nitrogen (BUN) (F (5,20) = 0.97; p = 0.46) and creatinine (F (5,20) = 0.60; p = 0.70) and the general organ damage marker lactate dehydrogenase (LDH) (F (5,20) = 0.62; p = 0.69) were also unchanged by ELP-VEGF injection (Supplementary Table 1 and Supplementary Fig. 2).

### Renal histological findings following ELP-VEGF treatment

**Figure 5:**
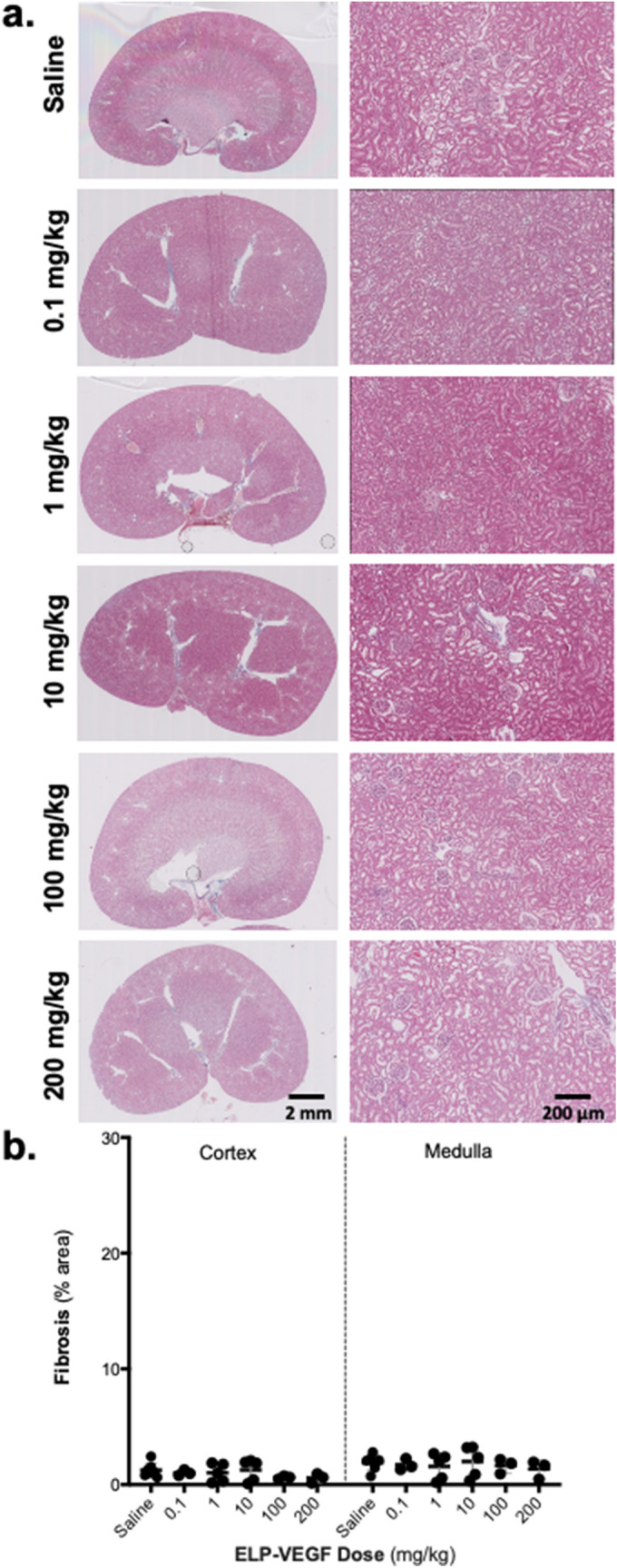
Renal Histology Following ELP-VEGF Therapy. **(a)** Renal sections taken from kidneys on Day 14 after ELP-VEGF injection were stained with Masson’s trichrome to assess renal fibrosis. (left bar = 2 mm, right bar = 200 µm). **(b)** Percentage fibrotic area was measured from each treatment group.

Renal histology was assessed on day 14 after ELP-VEGF injection via Masson’s trichrome staining. Gross assessment of whole-slice images and high magnification views of cortical and medullary sections showed no signs of glomerulosclerosis or overt tubular damage (Fig. 5a). Quantitation of the trichrome stain revealed minimal fibrosis present in all of the sections, none differing from control at any ELP-VEGF dose tested in either the renal cortex (F(5,20) = 0.83; p = 0.54) or medulla (F(5,20) = 0.26; p = 0.93, Fig. 5b).

### Effect of ELP-VEGF on renal microvascular density

**Figure 6:**
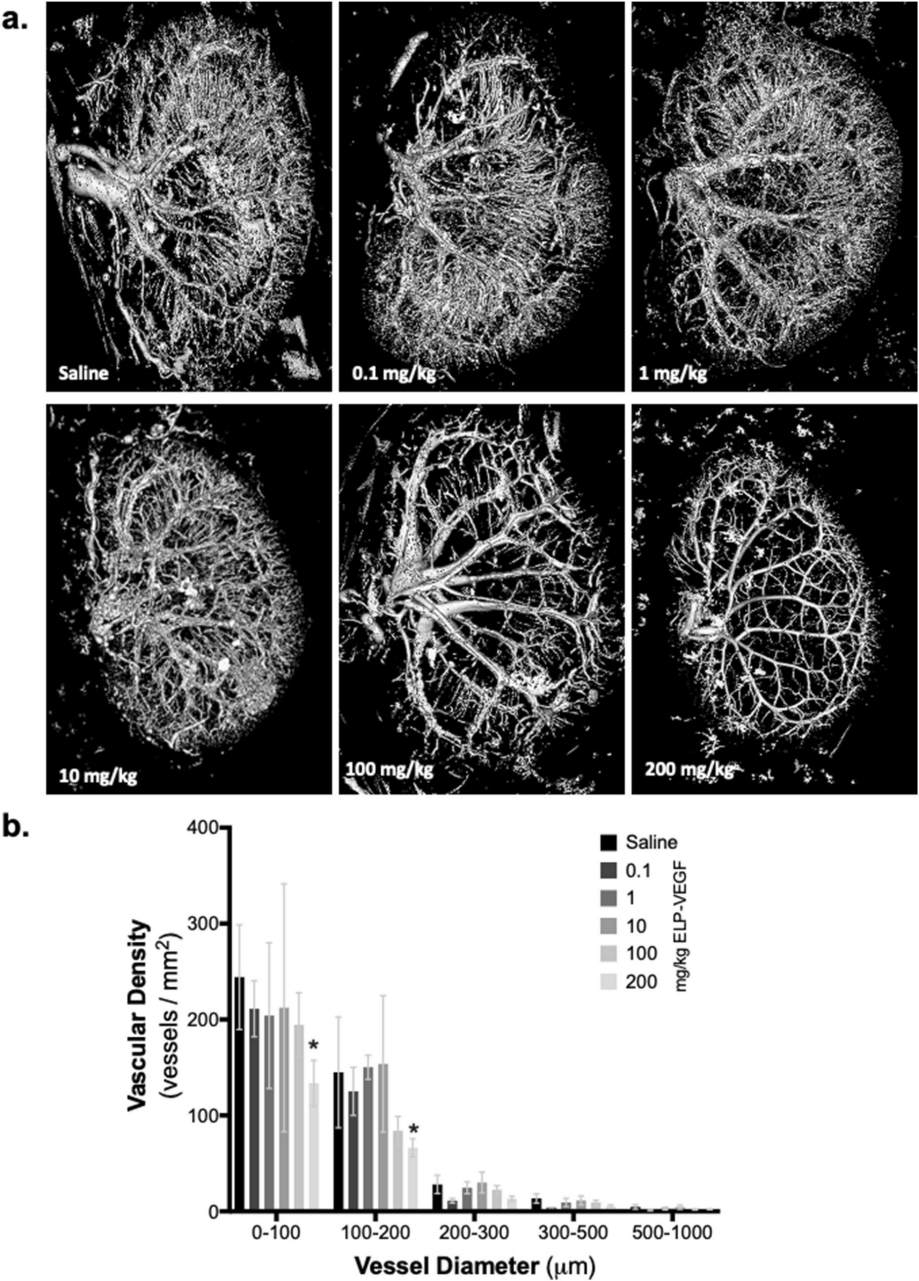
Renal Microvascular Density Following ELP-VEGF Therapy. **(a)** One kidney was perfused with Microfil on Day 14 following ELP-VEGF therapy, and microvascular density was assessed by μCT scanning. **(b)** Microvascular density of variously sized vessels was quantified from the reconstructed μCT images. * Statistically significant decrease relative to saline treated rats, p < 0.05, two-way ANOVA with post-hoc Dunnett correction for multiple comparisons.

Renal microvascular density was measured by μCT scanning on day 14 after ELP-VEGF administration. The entire renal vascular network was reconstituted in three dimensions (Fig. 6a). At lower doses (0.1 mg/kg, 1.0 mg/kg, 10.0 mg/kg), ELP-VEGF had no effect on vascular density across all vessel diameter ranges. However, at supratherapeutic doses (200 mg/kg), there was a significant decrease in renal vascular density in vessels ranging from 0–200 μm in diameter (F (4,95) = 96.54; p < 0.0001; Dunnett’s corrected p = 0.0007 and 0.03 for 0–100 μm and 100–200 μm vessel diameters, respectively) (Fig. 6b).

### Effects on ELP-VEGF on tumor progression and vascularization in a mouse breast cancer xenograft model

**Figure 7:**
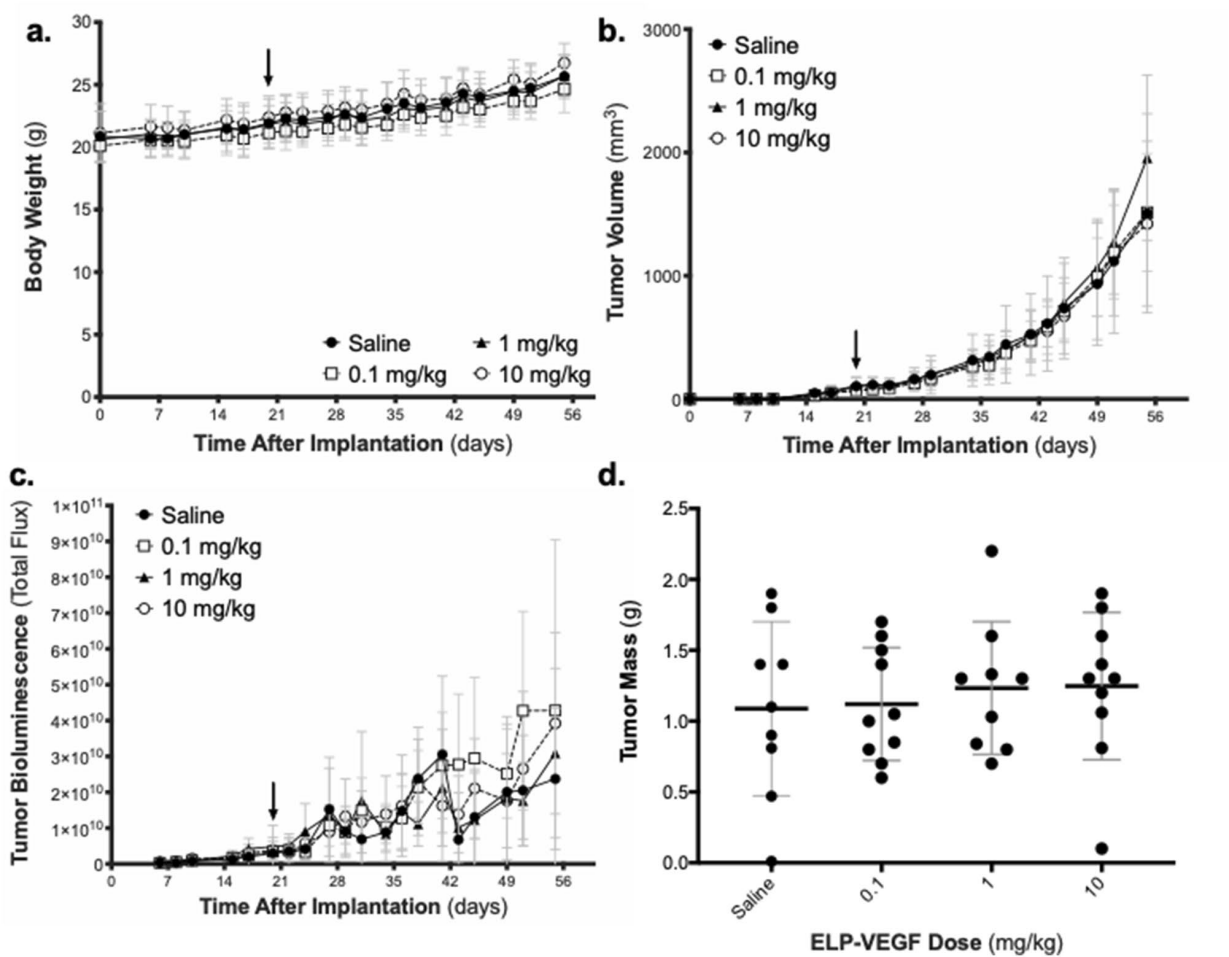
Effect of ELP-VEGF on Breast Tumor Progression. Body weight **(a)**, tumor volume **(b)**, and tumor bioluminescence **(c)** were tracked in nude mice bearing MDA-MB-231 triple negative breast tumor xenografts following a single IP bolus injection of ELP-VEGF (arrow). **(d)** Tumor mass was assessed at sacrifice five weeks after ELP-VEGF treatment.

In addition to direct toxicity, another concern for therapeutic angiogenesis strategies using VEGF supplementation is that they may exacerbate tumor growth in the event of a patient with an undiagnosed malignancy. In order to directly assess the effect of ELP-VEGF on tumor progression and vascularization, we utilized a mouse model of breast cancer. After establishment of orthotopic, triple negative human breast xenografts in athymic nude mice and administering ELP-VEGF in a single bolus, dose escalation paradigm, body weight and tumor progression were tracked. There was no change in the trajectory of body weight over time in the mice following ELP-VEGF injection at any dose (Fig. 7a, F (3,34) = 1.40; p = 0.26). There was also no change in the trajectory of tumor volume over time in the mice following ELP-VEGF injection at any dose as assessed by both caliper measurement (Fig. 7b, F (3,34) = 0.24; p = 0.87)) and tumor bioluminescence (Fig. 7c, F (3,34) = 1.44; p = 0.25). Tumor mass at euthanasia was also not different among treatment groups (Fig. 7d, F (3,34) = 0.24; p = 0.87).

**Figure 8:**
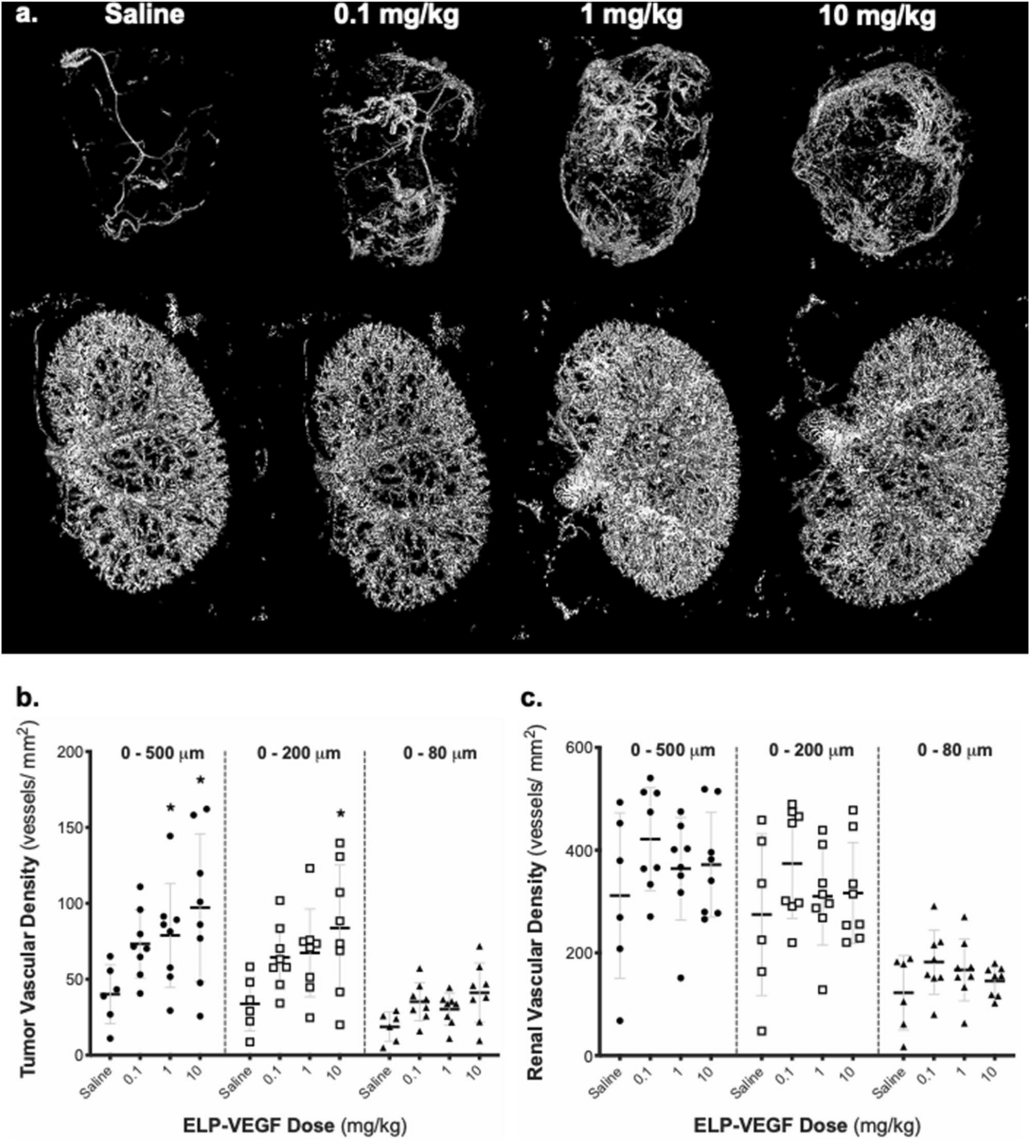
Effect of ELP-VEGF on Breast Tumor and Renal Vascular Density in Nude Mice. Vascular density in MDA-MB-231 breast tumors (**a**, top panel and quantified in **b**) and mouse kidneys (**a** bottom panel and quantified in **c**) was assessed five weeks after a single bolus ELP-VEGF injection at increasing doses by μCT scanning. * Statistically significant decrease relative to saline treated rats, p < 0.05, two-way ANOVA with post-hoc Dunnett correction for multiple comparisons.

Tumor and renal vascular density were assessed at euthanasia five weeks after ELP-VEGF treatment via μCT scanning (Fig. 8a). In tumors, ELP-VEGF at doses of 1 mg/kg and 10 mg/kg led to significant, dose dependent increases in microvascular density in vessels ranging from 0 to 500 μm (40.10 ± 19.48 to 78.88 ± 34.44 and 97.17 ± 48.65 vessels/mm^2^ respectively; p = 0.049 and p = 0.001, respectively) as well as 0–200 μm (10 mg/kg) in diameter (33.83 ± 17.82 to 83.77 ± 41.56 vessels/mm^2^, p = 0.006)(Fig. 8b). This increased vascular density was evident in cross sectional CT images (Supplementary Fig. 3), demonstrating increased vascularization of the tumor cores in mice treated with ELP-VEGF. However, there were no changes in renal vascular density at any vessel diameter in any ELP-VEGF dosing group (Fig. 8c, all Dunnett’s corrected p values > 0.1).

## Discussion

As a next step toward translating ELP-VEGF for treatment of chronic renal diseases, this study characterized the toxicological profile of ELP-VEGF and examined its effects on the progression of tumor growth and vascularity. ELP-VEGF administration had no long-term effect on blood pressure at previously determined therapeutic doses (0.1 and 1.0 mg/kg) or even at ten times the planned therapeutic dose. But at much higher doses (100–200 mg/kg), ELP-VEGF acutely, though not significantly, induced a transient reduction of blood pressure. This effect lasted less than thirty minutes, and the pressure returned back to baseline. Given VEGF’s ability to induce vascular nitric-oxide production^24^, it is likely that, at high doses, ELP-VEGF injection induced systemic vasodilation leading to transient hypotension. ELP-VEGF did not result in any plasma or urine markers of toxicity that were outside the normal limits, and renal histology was unaffected by ELP-VEGF treatment. There was a mild but statistically significant increase in urinary creatinine values in rats treated with 0.1 or 1.0 mg/kg ELP-VEGF, but not in the higher dose groups, on day 14 after treatment. The rats in this study were placed in a metabolic cage for a 24-h urine collection, from which creatinine was measured. It is plausible that, even though the rats had access to both their normal chow and fresh water ad libitum, some of the rats may have reduced or modulated their food and water intake. We postulate that this change in intake, instead of the therapeutic itself, is the most likely reason for the moderate increase in urine creatinine in the low to moderate dosing groups.

We previously showed that ELP-VEGF treatment caused a robust increase in urinary nitrate/nitrite levels when used as a therapeutic in a rat model of placental ischemia^33^, suggesting induction of renal NO signaling. Urinary nitrate/nitrite levels were not eleveated in the current study at any dose of ELP-VEGF. These differences are likely due to differences in treatment paradigms between the two studies. In the previous study, ELP-VEGF was administered continuously by osmotic pump for 5 days in pregnant SD rats at a dose of 1 – 10 mg/kg/day, and nitrate and nitrite levels were measured from spot urine samples taken at the time of harvest immediately at the end of the infusion period^33^. In contrast, in this study, urinary nitrate/nitrite levels were measured on day 7 or 14 after bolus ELP-VEGF injection. It is likely that induction of renal NO by ELP-VEGF is an acute and transient effect that was no longer present by the time we collected urine in this study.

ELP-VEGF caused a significant increase in GFR at the highest dose tested. Previous publications using ELP-VEGF in various models of renal disease have shown that at certain doses, ELP-VEGF increases GFR when compared to disease control^7, 8, 19^. We suspect that this increase is secondary to hyperfiltration caused by either an increase in renal blood flow due to vasodilation or an interaction of ELP-VEGF with either podocytes or another cell type causing a loosening of the basement membrane leading to increased filtration rate. Importantly, this increased filtration rate was not paired with albuminuria or nephrin shedding into the urine, suggesting that it is not indicative of a classical hyperfiltration renal injury^34, 35^. However, the increased GFR following 200 mg/kg ELP-VEGF was accompanied by a decrease in renal vascular density. Over-production of VEGF has been shown previously to be associated with deleterious renal effects. Eremina and Quaggin showed that conditional overexpression of VEGF-A in podocytes in adult transgenic mice led to collapsing glomerulopathy with hyperfused capillaries^36^. However, this finding was also associated with nephrotic-range proteinuria^37^, which was not seen in our high-dose ELP-VEGF treatment groups. Previous studies in rodent models of diabetes have suggested that elevated VEGF in a context of low nitric oxide (NO) may lead to direct vascular injury along with overactivation of the endothelium, vascular smooth muscle cell activation, and inflammation^38^. However, whether this mechanism is at play in our model is unclear, as one would not expect endogenous NO levels to be low in these healthy rats. Therefore, more experiments are needed to elucidate the exact mechanisms involved in the reduction of vascular density following these ultra-high, supra-therapeutic doses of ELP-VEGF.

We expanded the dose escalation toxicity studies with a dose escalation tumor progression study using a human breast cancer xenograft model to define the effects of ELP-VEGF therapy on an indolent, subclinical tumor. A single dosing treatment schedule was used to mimic the treatment schedule used in our previous swine efficacy studies^7, 18, 19, 39^. The intraperitoneal route was used to facilitate larger injection volumes, allowing us to use doses up to 10 mg/kg in mice. We were unable to test higher doses, as were used in the rat study, due to the small size of the mice and the limited injection volume possible in these animals. There was no effect of ELP-VEGF on tumor growth rate or final tumor mass even at ten times the highest therapeutic dose. The most significant finding from the xenograft study was an increase in tumor vascular density that was observed with increasing doses of ELP-VEGF. Tumor vascularity is critical for the growth and progression of tumors. It has been demonstrated that tumors with a rich vascular network of blood vessels grow rapidly and also have a higher propensity to metastasize^20^. However, it is important to note that during IVIS imaging and gross examination at the time of sacrifice, we did not observe metastasis of the xenograft tumor to any other organs in the mice. However, it is common for rapidly growing xenografts such as those used in this study to outgrow their blood supply and form necrotic cores. μCT analysis of the tumors in this study (Fig. 8 and Supplementary Fig. 3) confirmed that administration of ELP-VEGF increased the vascular density in the core of the tumors. Therefore, even though ELP-VEGF did not enhance the tumors’ overall growth rate and size, it is possible that, if allowed to continue further, the tumors in the ELP-VEGF treated mice would persist longer prior to “collapse” due to their better vascularized cores.

The increase in tumor vascular density after only a single ELP-VEGF treatment was somewhat surprising, but it is similar to the increases in renal microvascular density induced by a single ELP-VEGF treatment in injured, chronically hypoxic kidneys repeatedly seen in our swine models of renovascular disease and chronic kidney disease^7, 18, 19, 39^. The first consideration for possible explanations of this effect are the pharmacokinetics and tumor accumulation of ELP-VEGF. In a previous mouse study, we administered free VEGF and ELP-VEGF at a equimolar doses of 123 nmol/kg (equivalent to approximately 10 mg/kg ELP-VEGF)^9^. The fusion of ELP to VEGF slowed the plasma clearance rate two-fold, but ELP-VEGF still cleared with a fairly rapid half-life of 52.4 min. In a followup mouse study using a higher 20 mg/kg dose, ELP-VEGF showed a half-life of 6.2 h^17^. In a swine study of intra-renally administered ELP-VEGF at a dose of 1 mg/kg, the terminal half-life was 13.5 h using this unique route of delivery^18^. While ELP fusion clearly stabilizes VEGF and slows its plasma clearance, the protein is not likely to remain in the body for longer than a few days. Importantly, the increased vascular density in the ELP-VEGF treated groups were unique to the tumors and not seen in the kidneys. This could possibly stem from increased ELP-VEGF deposition in the tumors due to the enhanced permeability and retention (EPR) effect. Previous studies by the PI and others^40–42^ have demonstrated significant tumor accumulation of ELP proteins and have attributed part of the tumor accumulation to the EPR effect^43, 44^. These studies suggest that ELPs have the ability to indirectly target tumors through the EPR effect caused by the disorganized and leaky neovascularization that occurs within growing tumors. The localization of the increased vascularity solely to the growing tumors also suggests that there is some mechanism in the microenvironment of these tumors that increases and prolongs VEGF activation and induces subsequent angiogenesis. It is possible that these growing tumors are more “primed” for neovascularization in response to VEGF, which combined with ELP-VEGF accumulation in the tumors via the EPR effect may be playing a role in ELP-VEGF’s ability to stimulate prolonged vascular growths in these tumors. In the case of our renal injury models, data implicate both activation/homing of endothelial progenitor cells^19^ and programming of resident macrophages into a pro-angiogenic M2 phenotype^7^ as mechanisms by which angiogenesis can be induced from a single ELP-VEGF injection. In these renal injury models, a single bolus of ELP-VEGF effectively initiates a positive feedback loop that leads to prolonged endogenous VEGF production and activation of VEGF signaling in the diseased tissue. It is likely that similar mechanisms are at play in the tumor microenvironment. In summary, the data from our xenograft experiment suggest that a single administration of ELP-VEGF has the potential to significantly increase the vascular density of pre-existing tumors in a dose-dependent fashion, which could potentially lead to unfavorable outcomes. More experiments are needed to elucidate the exact mechanism of this effect, but given these results, it is possible that therapy with ELP-VEGF could be contraindicated in the setting of preexisting malignancies due to its effects on vascular growth.

Given the entirety of the results from both the toxicology and xenograft studies, we concluded that, at previously established therapeutic doses^18, 19^, ELP-VEGF had minimal to no adverse effects on renal function, blood pressure, vital organ function, or tumor size. However, significant changes in the vascularity of tumors were seen at therapeutic and supratherapeutic doses. Also, at supratherapeutic doses (200–2000 times the therapeutic dose), ELP-VEGF caused both renovascular and systemic hemodynamic changes.

One potential limitation of these studies is the use of rodent models. While we have not yet measured the affinity of ELP-VEGF for the rodent VEGF receptors, we have shown previously that ELP-VEGF (which includes the human VEGF-A_121_ isoform) is active in rodents^33^. Additionally, VEGF-A and its most active receptor, Flk-1, are highly conserved across all species used in our preclinical development of ELP-VEGF (Supplementary Fig. 4). Brozzo et al. previously solved the crystal structure of human VEGF-A bound to immunoglobulin-like domains 2 and 3 of Flk-1, identifying all residues that make contact between the cytokine and the receptor^45^. All six residues in VEGF-A that make contact with receptor (highlighted in red in the adaptation of Brozzo’s crystal structure in Supplementary Fig. 4a) are 100% conserved across mouse, rat, swine, and human (Supplementary Fig. 4b), and six of the seven receptor residues involved in these contacts are conserved (highlighted in blue in Supplementary Fig. 4a, alignment shown in Supplementary Fig. 4c). The only exception is Flk-1 residue 313, which is leucine in human, rat, and swine, but is arginine in the mouse. This very high conservation, combined with our data demonstrating ELP-VEGF activity in all of the above species, strongly suggests that the preclinical models we have chosen are valid for initial assessment of ELP-VEGF efficacy and toxicity.

With regard to the μCT analysis, we acknowledge that it is possible that due to limitations in the resolution of the μCT, some of the smallest microvessels were not included in the quantification of microvascular density. Additional experimental platforms may be needed (e.g. tissue sections stained with CD-31 to quantify capillaries, as previously shown^5^, which would be addressed in future studies as the tumors in this study were not amenable to histological staining after Microfil infusion). Nevertheless, based on the similar conditions of preparation and scanning of the samples and the fact that quantification was performed in a blinded fashion, it is likely that any differences due to vessels smaller than the μCT detection limit would not significantly skew the results.

Finally, we recognize that long-term effects of ELP-VEGF may not have been uncovered during the 14-day time frame of this toxicology study or the five-week time frame of the tumor progression study. Repeated dosing could also affect the toxicological profile, and it is possible that the toxicology could differ in subjects with renal injury, potentially necessitating future studies. For the tumor study, we chose to begin with a breast cancer model because it is the most prevalent cancer in females and thus likely to be encountered among the patient population also at risk for renal disease. But we acknowledge that other cancer models, including using male subjects and especially renal cancer models, will need to be tested in the future in order to fully understand the effects of ELP-VEGF in the setting of oncological diseases. Even with these caveats, this study demonstrated that ELP-VEGF has a high safe-dosing window in subjects without cancer as a contraindication. These results support the continued development of ELP-VEGF as an agent for therapeutic angiogenesis to treat ischemic kidney diseases.

## Materials and methods

### Cloning, purification, and characterization of ELP-VEGF

A fusion protein containing ELP (160 repeats of the pentapeptide VPGxG, where x is valine, glycine, or alanine in a 1:7:8 ratio) and human VEGF-A_121_ (ELP-VEGF) was purified via recombinant expression in *E. coli* as described in^9^. Protein purity and VEGF identity were confirmed with SDS PAGE and Western Blot analysis, respectively. The transition temperature of ELP-VEGF was determined as a function of ELP-VEGF concentration in physiological phosphate buffered saline (PBS) and as a function of sodium chloride concentration in phosphate buffer (10uM ELP-VEGF) plus sodium chloride varying from 25 to 700 mM. Aggregation of ELP-VEGF under these conditions was detected by monitoring the optical density at 350 nm (OD_350_) while increasing the temperature at a rate of 0.5 °C / minute in a UV/Visible spectrophotometer with a temperature-controlled Peltier block (Cary). Raw turbidity data were converted to a percentage of the maximum OD_350_ in order to account for concentration differences and display all curves on the same plot. The transition temperature (T_t_) was defined as the temperature at which a maximum was observed in a plot of the first derivative of the aggregation curve.

### Animal use

All protocols were approved by the University of Mississippi Medical Center Institutional Animal Care and Use Committee and followed the National Institutes of Health Guidelines for the Care and Use of Laboratory Animals. SD rats (Charles River) were received and acclimated to the animal facilities for at least seven days prior to experimentation. Athymic nude mice (Charles River) were received and acclimated similarly in a pathogen-free housing facility. Rats and mice were maintained on a 12:12 light:dark cycle, maintained at constant 23 °C, and provided food at water ad libitum. All animal experiments in this study were conducted in compliance with the ARRIVE guidelines.

### Dose escalating toxicology study

A cohort of SD rats (female, n = 18) were implanted with telemetry devices (PA-C10, Data Sciences Inc.) in sterile conditions under 3% isoflurane anesthesia and given a subcutaneous injection of carprofen (5 mg/kg). While under anesthesia, rats were also implanted with a jugular catheter, composed of V-3 and V-1 tubing (Scientific Commodities), which was routed subcutaneously and externalized dorsally where it was attached to a jugular venous access port (VABR1B/22, InstechLabs) and secured with 4–0 silk suture. Mean arterial pressure (MAP) was measured through implanted telemeters. Jugular ports were flushed with 30% heparinized saline three times per week to maintain cannula patency. MAP measurements were taken every 30 min after implantation for 48 h to allow MAP to return to baseline. After 48 h of recovery from surgery, rats received an injection with a dose of either ELP-VEGF (0.1, 1.0, or 10.0 mg/kg) or saline administered through the jugular venous access port, and MAP was measured every 5 min after injection and then continually measured every 30 min for 14 days.

Due to the absence of significant changes in MAP seen with the initial injection doses, we added a second cohort of SD rats (female, n = 8) which were implanted with catheters composed of V-3 and V-1 tubing ( Scientific Commodities ) in both the left common carotid artery and the right jugular vein under the same conditions as discussed above. Rats in this cohort were connected to direct blood pressure measurement devices (ADInstruments) and were allowed to acclimate for one hour. Rats then received an injection of ELP-VEGF (100 mg/kg, 200 mg/kg) or saline through the jugular catheter, and MAP was measured continuously for 60 min. Rats were then removed from blood pressure monitors and housed individually.

Daily body weight measurements were taken during the 14 days after injection in both cohorts. At the end of the 14-day period, blood samples were collected in EDTA coated vials, the left kidney was ligated and removed for histological analysis, and the abdominal aorta was catheterized and used to perfuse the renal vasculature of the right kidney with heparinized saline followed by Microfil (MV122, Flow Tech). The right kidney was then removed and stored at 4 °C for 24 h to allow polymerization of the Microfil and scanned using a benchtop micro-computed tomography (μCT) as described below.

### Renal function measurements

Glomerular Filtration Rate (GFR) was measured at three separate times throughout the study. Baseline GFR was measured the day prior to ELP-VEGF injection, and repeat GFR measurements were made on days 7 and 14 after ELP-VEGF injection. At the time of measurement, a dorsal area of hair was shaved, and transdermal probes (MediBeacon)^46^ were attached to the shaved area and immobilized with a rubber harness under 3% isoflurane. Jugular access ports were flushed with heparinized saline, and a bolus of FITC-Sinistrin (50 mg/kg, Fresenius-Kabi) was injected through the port. The half-life (t_1/2_) of the clearance was measured by a transdermal probe (MediBeacon) and used to calculate GFR using the following equation:

$$GFR \, \left( {\text{mL/ min per 100 g BW}} \right) = \left( {31.26 \, \left[ {\text{mL/100 g BW}} \right]/t1/2} \right)$$

^47, 48^. Additionally, rats were placed in individual metabolic cages for 24 h 3 times throughout the study (Baseline, Day 7, Day 14), and 24-h urine samples were collected. Assays were run on the urine to analyze albuminuria (Abcam) and nephrin excretion (Ethos Biosciences) via ELISA, total nitric oxide via the Griess reaction (Cayman), and urine creatine levels (VetAxcel Chemical Analyzer) according to manufacturers’ instructions.

### Renal histology

Kidneys were fixed for 48 h in Histofix (BioRad), embedded in paraffin, and cut into 10 μm sections captured onto slides by the Histology Core Facility at the Univesity of Mississippi Medical Center. Slides were then stained with trichrome and preserved with Permount (Thermo-Fisher) and a coverslip. Whole-slide images were collected by brightfield slide scanning at 20 × magnification by the University of Mississippi Medical Center Pathology Core Facility. For analysis, 10 fields were selected from consistent regions of the kidney on each slide (5 cortical and 5 medullary) and analyzed for % area fibrosis, as described^5^. Analysis was conducted by J.P.W., who was blinded to the treatment group of each image.

### Blood chemistries

Blood collected during euthanasia (14 days after ELP-VEGF treatment) was spun at 13,000 *g*, and plasma was collected and stored at − 80 °C. Plasma was assayed for alanine aminotransferase (ALT), aspartate transaminase (AST), total bilirubin, albumin, creatine, blood urea nitrogen (BUN), and lactate dehydrogenase (LDH) levels using a VetAxcel Chemical Analyzer via the University of Mississippi Medical Center Analytical and Assay Core. The core employees were blinded to the treatment groups.

### Dose escalating tumor promotion study

MDA-MB-231 human triple negative breast cancer cells stably expressing luciferase and red fluorescent protein were purchased from Amsbio (SC041) and maintained according to vendor recommendations. To establish tumors, 2 × 10^6^ cells were suspended in 200 μl of Matrigel Matrix (Corning 356234). Cell suspensions were implanted into 40 anesthetized female athymic nude mice by mammary fat pad injection using a 23-gauge needle. Three times per week, subjects were evaluated for tumor growth using a caliper to measure tumor size across two perpendicular axes. Tumor volume was calculated using the formula:

$$V \, = \, \left( {w^{2} x \, l} \right)/2$$

where *w* represents the width of the shortest axis and *l* represents the length of an axis perpendicular to *w*. Tumor volumes were measured by an observer who was blinded to the treatment groups (S.P.B). Additionally, tumor progression was analyzed by tracking bioluminescence three times per week using an IVIS Spectrum (Perkin Elmer) after intraperitoneal injection of luciferin (XenoLight D-Luciferin—K + Salt Bioluminescent Substrate, Perkin Elmer). Three weeks after tumor implantation, the subjects were randomized into 4 groups (n = 10 mice per group) to achieve an equal average tumor volume in each group, then treated with saline or 0.1, 1.0, or 10.0 mg/kg of recombinant ELP-VEGF via intraperitoneal injection. Body weight, tumor volume, and tumor bioluminescence measurements continued as above for five weeks after injection. The subjects were then sacrificed by transcardial perfusion with heparinized saline followed by Microfil (MV122, Flow Tech), and the cadavers were held overnight at 4 °C to allow polymerization of Microfil. Tumors and tissues of interest were harvested the following day, major organs were assessed grossly for any overt signs of tumor formation, and tumors were weighed. Microfil—perfused tumors and tissues were then analyzed using μCT imaging to assess vascular density as described below.

### μCT analysis of tumor and renal vascular density

Micro CT scanning was conducted using a SkyScan 1076 system (Bruker, Belgium). Each sample was equally positioned in the scanning bed and scanned at 0.3° angular increments, as previously described^5, 18, 49, 50^. X-ray transmission images were acquired in each angle of view at a resolution of 18 µm, digitized to 16 bits gray scale, reconstructed using a filtered back-projection algorithm, and displayed on a computer workstation by volume rendering for display and analysis of microvascular density using the ANALYZE (Biomedical Imaging Resource, Mayo Clinic, Rochester, MN) software package. Microvascular spatial density was calculated in a blinded fashion by semi-automatically counting microvessels in selected regions of interest using an “Object Counter” program that is part of the ANALYZE software package. Each sample was tomographically divided into 15 slices following the z axis at equal intervals, from top to bottom of the sample. After manually setting a threshold for intensity, which is determined by the presence of Microfil inside the vessels that are observed as circumferential structures of different diameters in each of the 15 2D z axis sections, the “Object Counter” calculated the number of those structures per microvascular diameter in each slice. The microvascular density per area of the region of interest (each tomographically generated slice) of tumor and renal microvessels of diameters between 0 and 500 µm were progressively calculated in each level using the ANALYZE software, and the total number of all the slices averaged. Analysis of μCT data was performed by a team member (A.R.C.) who was blinded to the treatment groups.

### Statistical analysis

Results from treatment groups are expressed as mean ± standard deviation. Differences among treatment groups in blood pressure and body weight in the rat study and tumor progression data in the mouse study were determined by two-way repeated measures ANOVA with factors for time and dose. Post-hoc multiple comparisons were performed using Tukey’s multiple comparison test. GFR and renal metabolites were compared using a two-way ANOVA with factors for dose and time point, and the Dunnett correction for multiple comparisons was used to assess statistical significance at each time point. Terminal data for % renal fibrosis and individual plasma metabolites in the rat study and tumor mass in the mouse study were compared using a one-way ANOVA, with the Dunnett correction for multiple comparisons. Micro-CT data were compared using a two-way ANOVA with factors for dose and vessel diameter, and the Dunnett correction for multiple comparisons was used to assess effects of ELP-VEGF dose within each vessel size group. Statistical significance was accepted for *p* ≤ *0.05* for all analyses. Statistics were calculated using GraphPad Prism.

## Supplementary Information

Supplementary Figures.

Supplementary Tables.

## Data Availability

All datasets generated during and/or analyzed during the current study are available from the corresponding author on reasonable request.
