# Supplementary material for: A dose-escalating toxicology study of the candidate biologic ELP-VEGF
Source: Sci Rep. 2021 Mar 18;11:6216. doi: 10.1038/s41598-021-85693-6 (PMC7973730; doi:10.1038/s41598-021-85693-6)
Supplement: Supplementary file 1 — Supplementary Figures. [file 41598_2021_85693_MOESM1_ESM.pptx]

## Slide 1
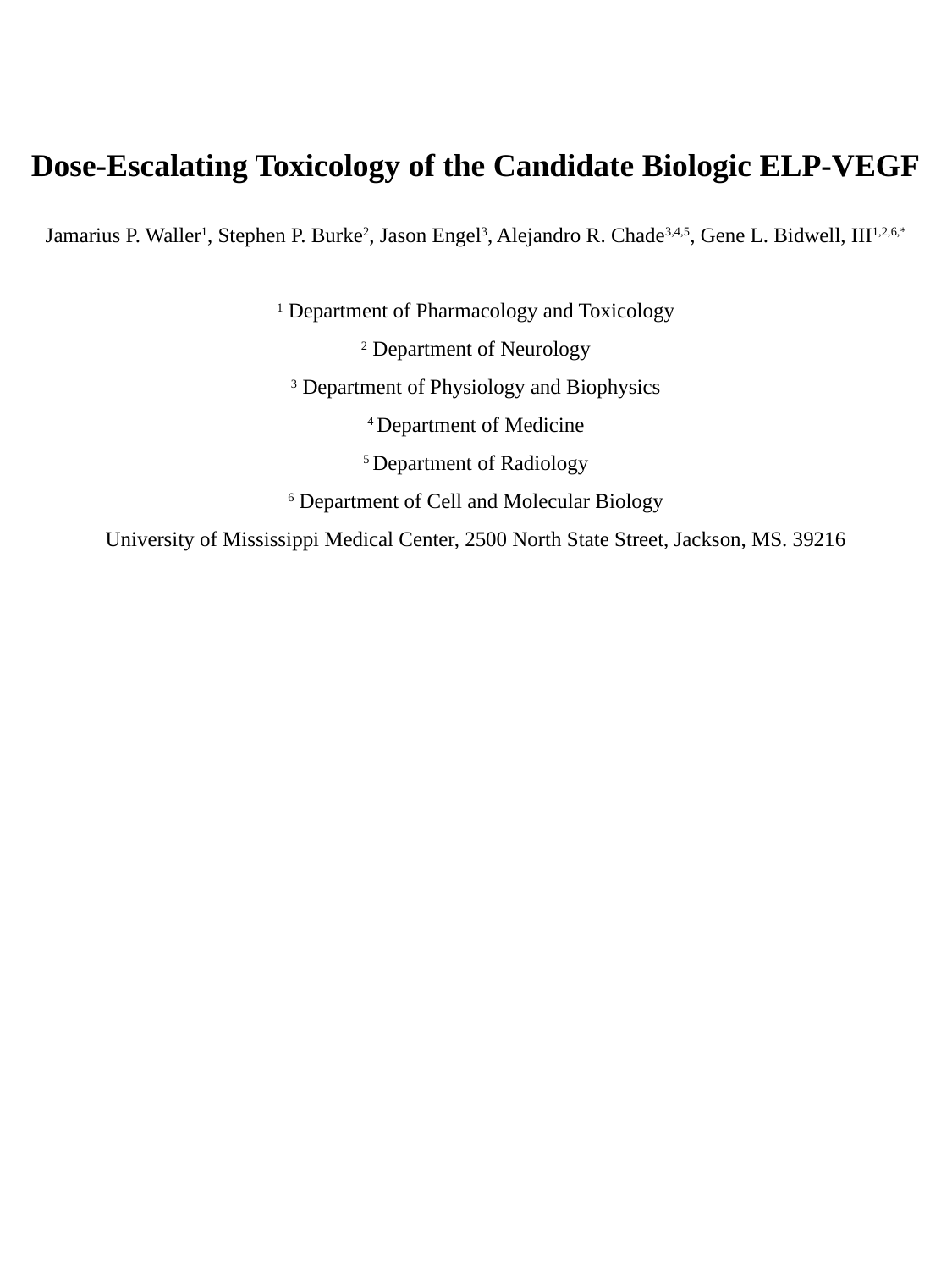

Dose-Escalating Toxicology of the Candidate Biologic ELP-VEGF
Jamarius P. Waller1, Stephen P. Burke2, Jason Engel3, Alejandro R. Chade3,4,5, Gene L. Bidwell, III1,2,6,*
1 Department of Pharmacology and Toxicology
2 Department of Neurology
3 Department of Physiology and Biophysics
4 Department of Medicine
5 Department of Radiology
6 Department of Cell and Molecular Biology
University of Mississippi Medical Center, 2500 North State Street, Jackson, MS. 39216

## Slide 2
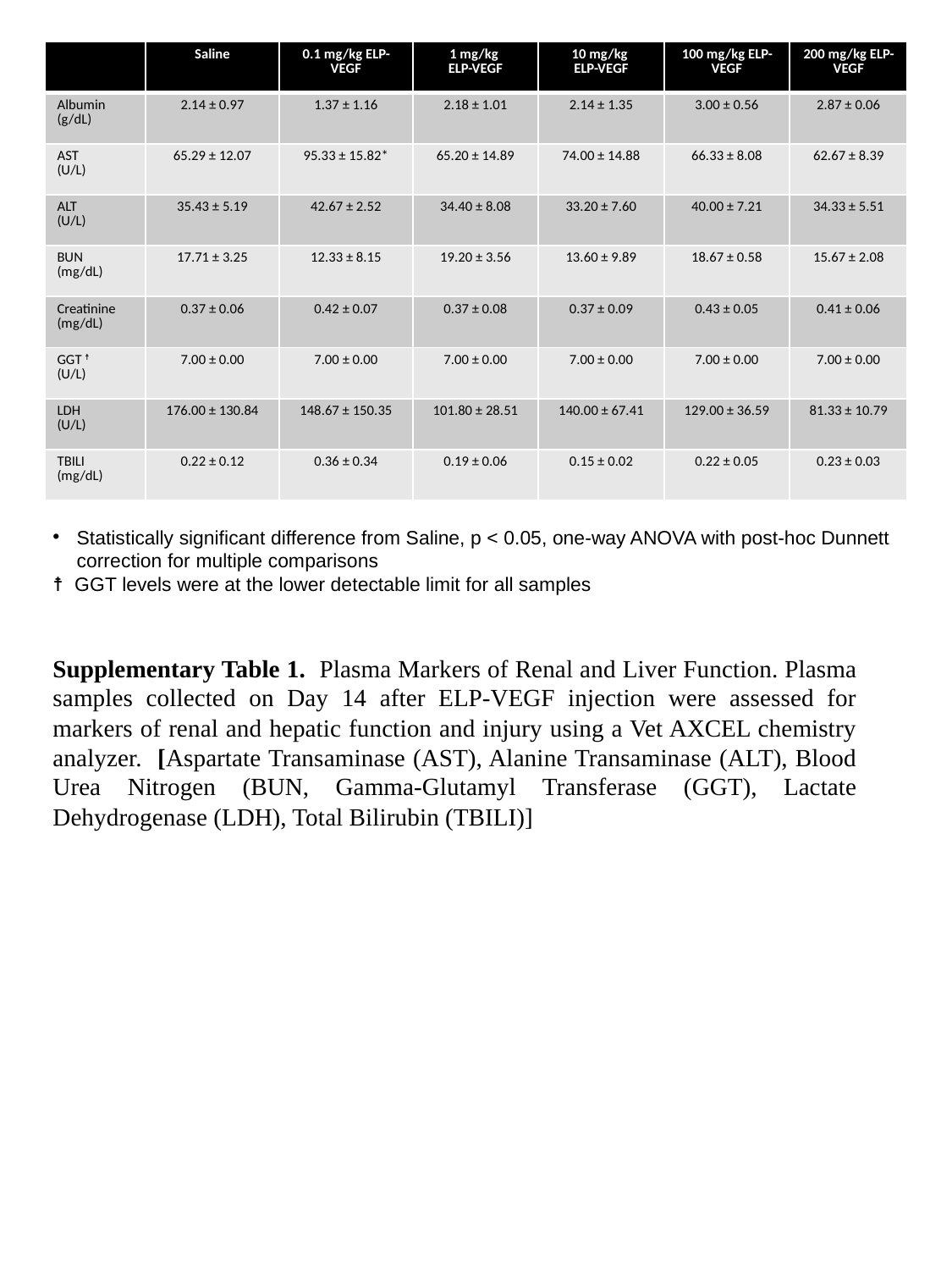

| | Saline | 0.1 mg/kg ELP-VEGF | 1 mg/kg ELP-VEGF | 10 mg/kg ELP-VEGF | 100 mg/kg ELP-VEGF | 200 mg/kg ELP-VEGF |
| --- | --- | --- | --- | --- | --- | --- |
| Albumin (g/dL) | 2.14 ± 0.97 | 1.37 ± 1.16 | 2.18 ± 1.01 | 2.14 ± 1.35 | 3.00 ± 0.56 | 2.87 ± 0.06 |
| AST (U/L) | 65.29 ± 12.07 | 95.33 ± 15.82\* | 65.20 ± 14.89 | 74.00 ± 14.88 | 66.33 ± 8.08 | 62.67 ± 8.39 |
| ALT (U/L) | 35.43 ± 5.19 | 42.67 ± 2.52 | 34.40 ± 8.08 | 33.20 ± 7.60 | 40.00 ± 7.21 | 34.33 ± 5.51 |
| BUN (mg/dL) | 17.71 ± 3.25 | 12.33 ± 8.15 | 19.20 ± 3.56 | 13.60 ± 9.89 | 18.67 ± 0.58 | 15.67 ± 2.08 |
| Creatinine (mg/dL) | 0.37 ± 0.06 | 0.42 ± 0.07 | 0.37 ± 0.08 | 0.37 ± 0.09 | 0.43 ± 0.05 | 0.41 ± 0.06 |
| GGT ☨ (U/L) | 7.00 ± 0.00 | 7.00 ± 0.00 | 7.00 ± 0.00 | 7.00 ± 0.00 | 7.00 ± 0.00 | 7.00 ± 0.00 |
| LDH (U/L) | 176.00 ± 130.84 | 148.67 ± 150.35 | 101.80 ± 28.51 | 140.00 ± 67.41 | 129.00 ± 36.59 | 81.33 ± 10.79 |
| TBILI (mg/dL) | 0.22 ± 0.12 | 0.36 ± 0.34 | 0.19 ± 0.06 | 0.15 ± 0.02 | 0.22 ± 0.05 | 0.23 ± 0.03 |
Statistically significant difference from Saline, p < 0.05, one-way ANOVA with post-hoc Dunnett correction for multiple comparisons
☨ GGT levels were at the lower detectable limit for all samples
Supplementary Table 1. Plasma Markers of Renal and Liver Function. Plasma samples collected on Day 14 after ELP-VEGF injection were assessed for markers of renal and hepatic function and injury using a Vet AXCEL chemistry analyzer. [Aspartate Transaminase (AST), Alanine Transaminase (ALT), Blood Urea Nitrogen (BUN, Gamma-Glutamyl Transferase (GGT), Lactate Dehydrogenase (LDH), Total Bilirubin (TBILI)]
