# Supplementary material for: A dose-escalating toxicology study of the candidate biologic ELP-VEGF
Source: Sci Rep. 2021 Mar 18;11:6216. doi: 10.1038/s41598-021-85693-6 (PMC7973730; doi:10.1038/s41598-021-85693-6)
Supplement: Supplementary file 2 — Supplementary Tables. [file 41598_2021_85693_MOESM2_ESM.pptx]

## Slide 1
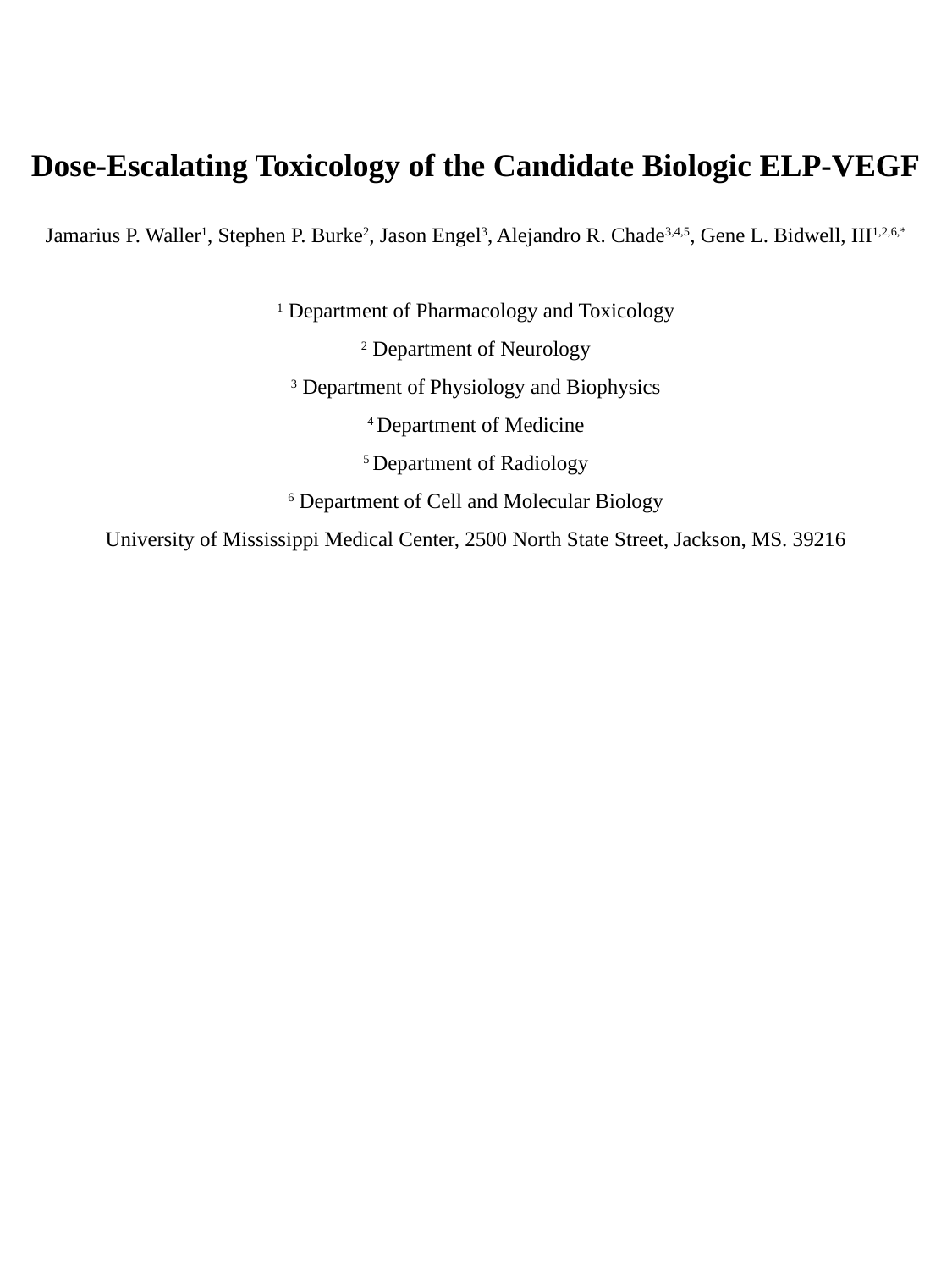

Dose-Escalating Toxicology of the Candidate Biologic ELP-VEGF
Jamarius P. Waller1, Stephen P. Burke2, Jason Engel3, Alejandro R. Chade3,4,5, Gene L. Bidwell, III1,2,6,*
1 Department of Pharmacology and Toxicology
2 Department of Neurology
3 Department of Physiology and Biophysics
4 Department of Medicine
5 Department of Radiology
6 Department of Cell and Molecular Biology
University of Mississippi Medical Center, 2500 North State Street, Jackson, MS. 39216

## Slide 2
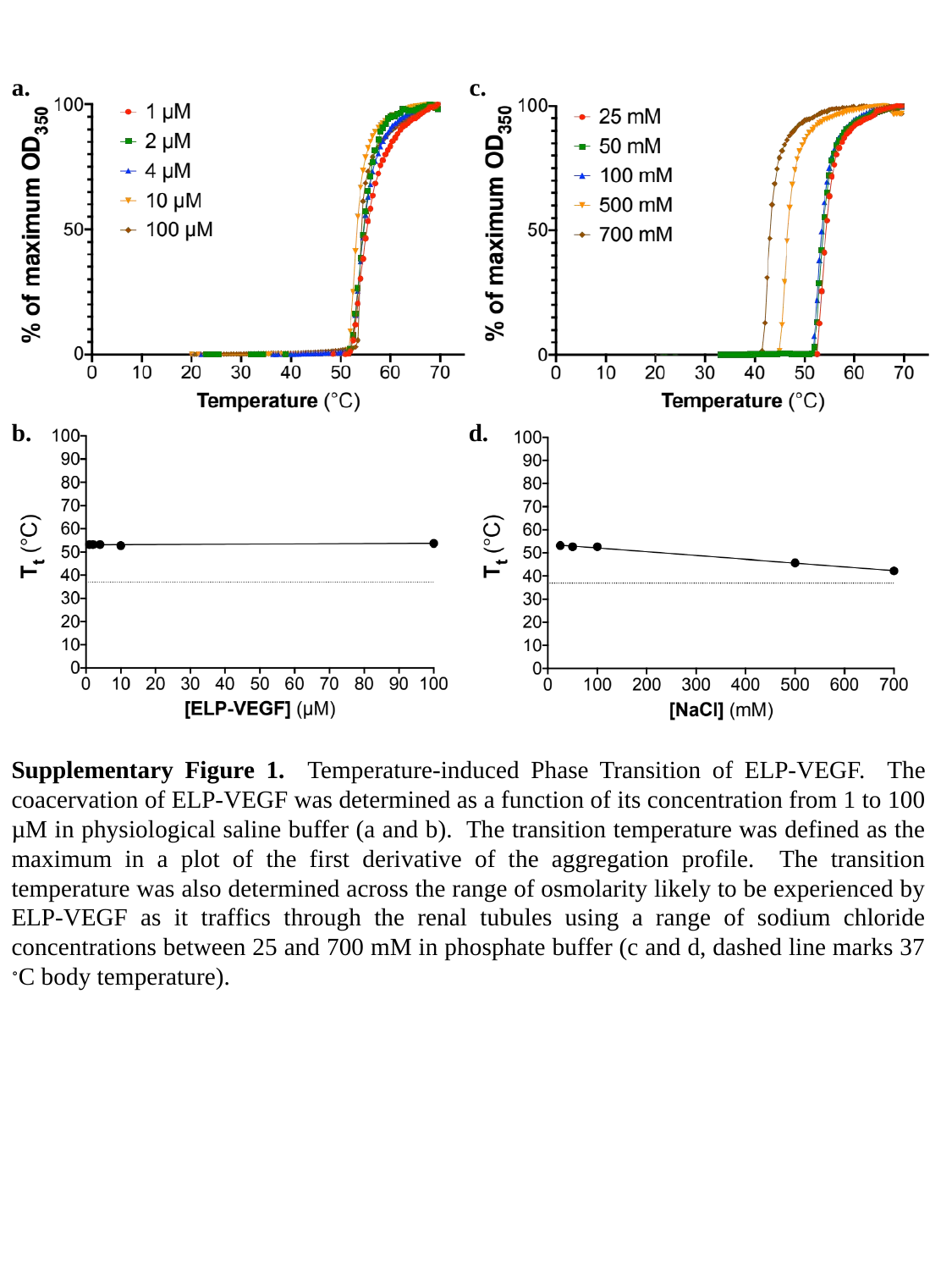

a.
c.
b.
d.
Supplementary Figure 1. Temperature-induced Phase Transition of ELP-VEGF. The coacervation of ELP-VEGF was determined as a function of its concentration from 1 to 100 µM in physiological saline buffer (a and b). The transition temperature was defined as the maximum in a plot of the first derivative of the aggregation profile. The transition temperature was also determined across the range of osmolarity likely to be experienced by ELP-VEGF as it traffics through the renal tubules using a range of sodium chloride concentrations between 25 and 700 mM in phosphate buffer (c and d, dashed line marks 37 ∘C body temperature).

## Slide 3
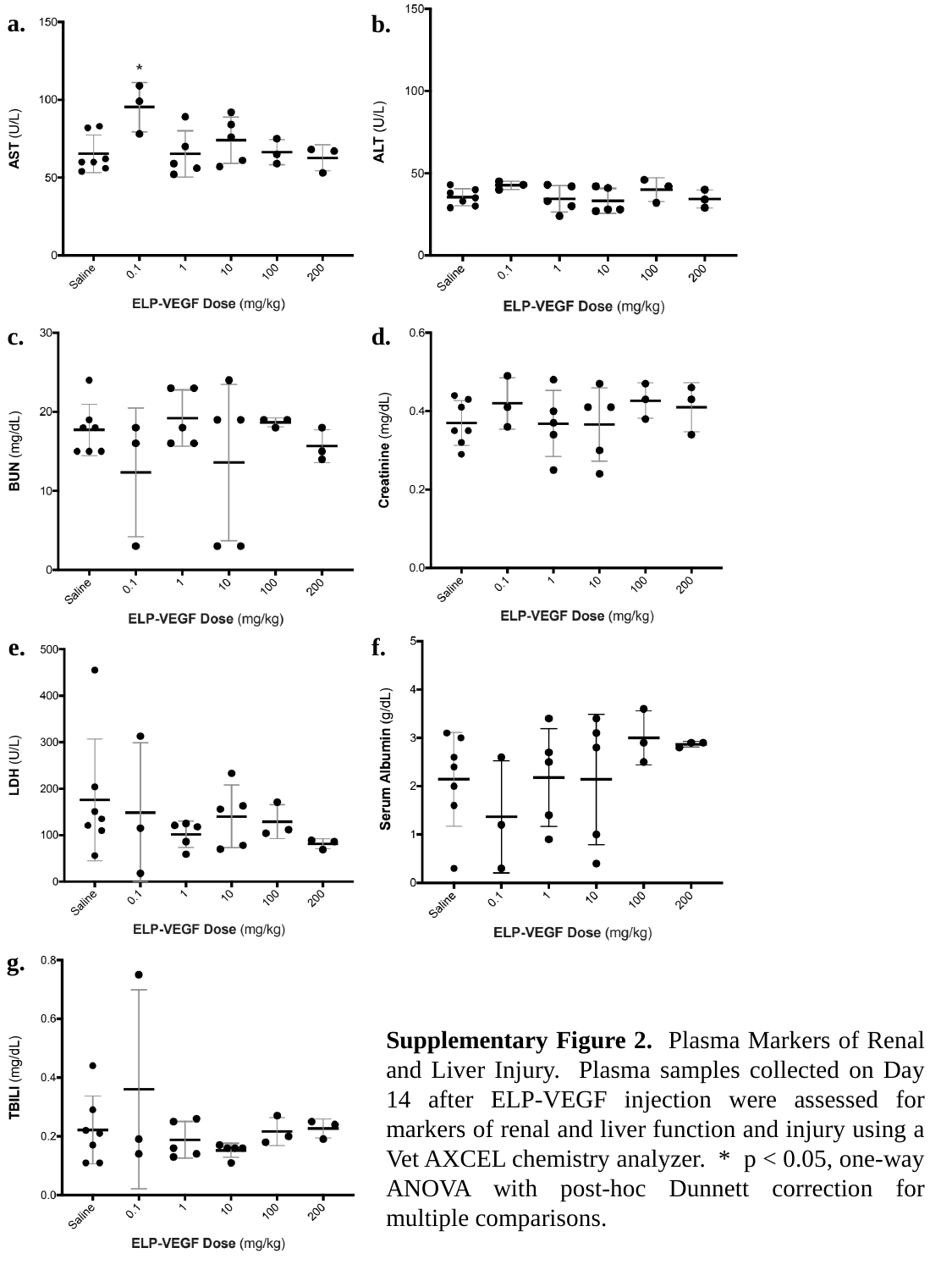

a.
b.
c.
d.
e.
f.
g.
Supplementary Figure 2. Plasma Markers of Renal and Liver Injury. Plasma samples collected on Day 14 after ELP-VEGF injection were assessed for markers of renal and liver function and injury using a Vet AXCEL chemistry analyzer. * p < 0.05, one-way ANOVA with post-hoc Dunnett correction for multiple comparisons.

## Slide 4
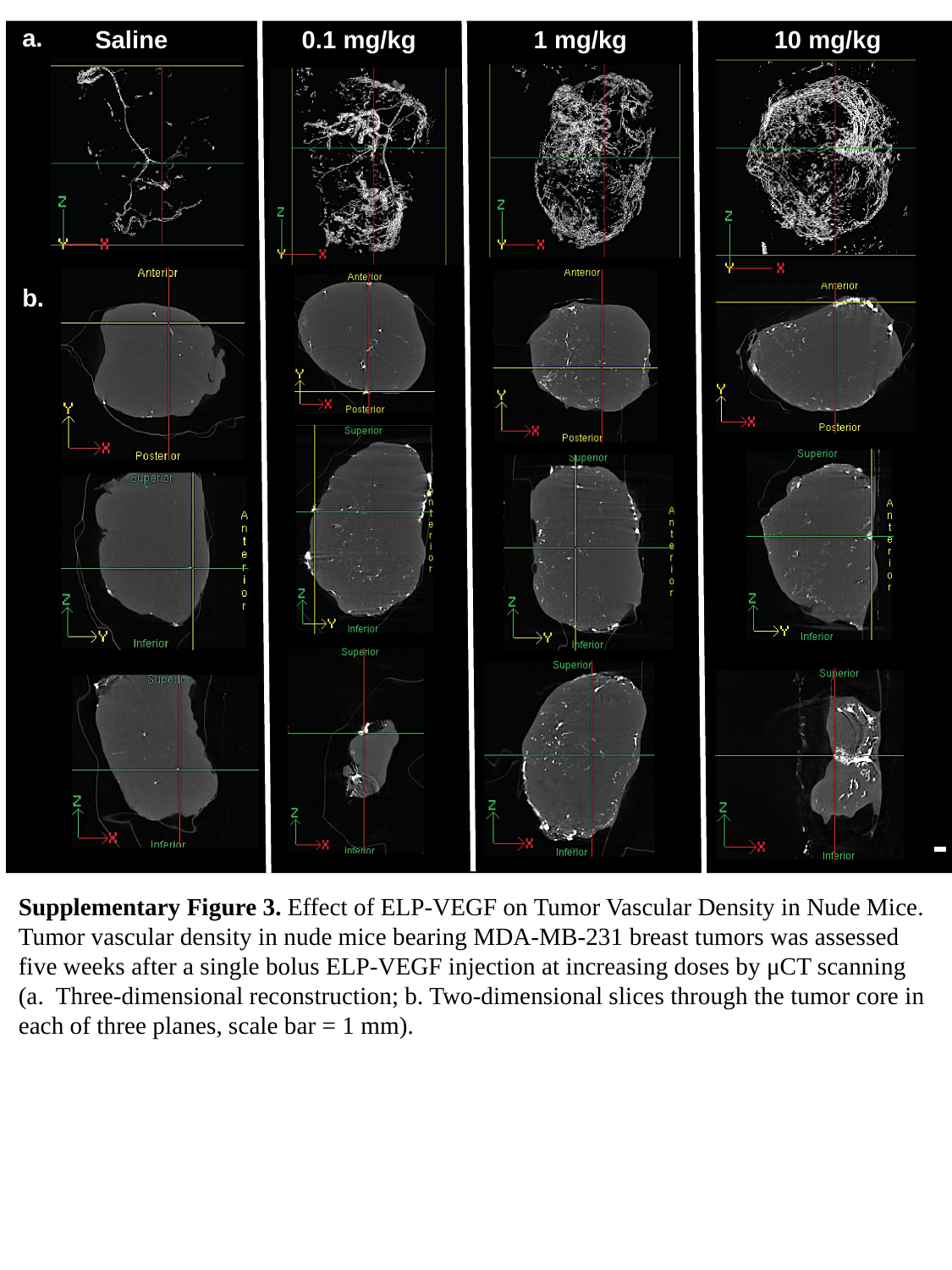

a.
Saline
0.1 mg/kg
1 mg/kg
10 mg/kg
b.
Supplementary Figure 3. Effect of ELP-VEGF on Tumor Vascular Density in Nude Mice. Tumor vascular density in nude mice bearing MDA-MB-231 breast tumors was assessed five weeks after a single bolus ELP-VEGF injection at increasing doses by μCT scanning (a. Three-dimensional reconstruction; b. Two-dimensional slices through the tumor core in each of three planes, scale bar = 1 mm).

## Slide 5
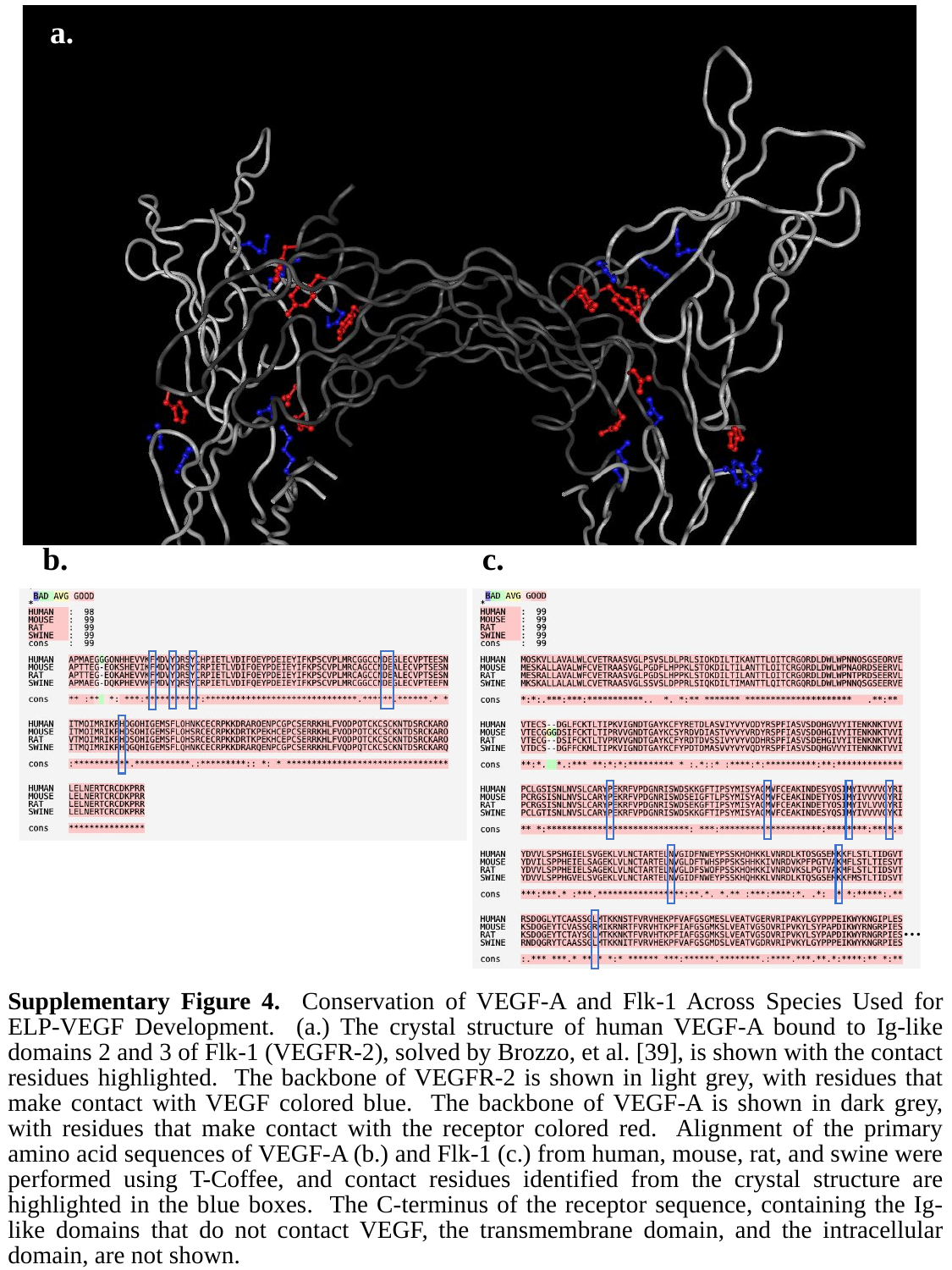

…
a.
b.
c.
Supplementary Figure 4. Conservation of VEGF-A and Flk-1 Across Species Used for ELP-VEGF Development. (a.) The crystal structure of human VEGF-A bound to Ig-like domains 2 and 3 of Flk-1 (VEGFR-2), solved by Brozzo, et al. [39], is shown with the contact residues highlighted. The backbone of VEGFR-2 is shown in light grey, with residues that make contact with VEGF colored blue. The backbone of VEGF-A is shown in dark grey, with residues that make contact with the receptor colored red. Alignment of the primary amino acid sequences of VEGF-A (b.) and Flk-1 (c.) from human, mouse, rat, and swine were performed using T-Coffee, and contact residues identified from the crystal structure are highlighted in the blue boxes. The C-terminus of the receptor sequence, containing the Ig-like domains that do not contact VEGF, the transmembrane domain, and the intracellular domain, are not shown.
